# Supplementary material for: Cumulative cultural evolution and mechanisms for cultural selection in wild bird songs
Source: Nat Commun. 2022 Jul 11;13:4001. doi: 10.1038/s41467-022-31621-9 (PMC9276793; doi:10.1038/s41467-022-31621-9)
Supplement: Supplementary file 1 — Supplementary Information [file 41467_2022_31621_MOESM1_ESM.pdf]

Supplementary Table 1. Values of conformity (β), selection (σ), and evaluation of models with and without frequency-dependent and direct selection.

	Conformity (β)	Selection (σ)	MLE	AIC	ΔAIC	weight
**Direct Selection**	1	1.70	-20.6	45.2	0	0.500
**Full model**	0.99	1.71	-20.6	45.2	0	0.500
**Frequency-dependent selection**	0.74	1	-54.4	112.8	67.6	0.000
**Drift**	1	1	-61.6	127.2	82.0	0.000

Each of these models considered songs with both high note clusters and click trains to have a blended trait and assumed that 2 innovators singing the blended trait joined the population in 1983. For the drift model, the effects of frequency-dependent and direct selection were excluded by setting β and σ to 1 (neutral). The value of β was fixed at 1 for the direct selection model, and the value of σ was fixed at 1 for the frequency-dependent selection model. Other parameters were varied from 0.5 to 2.0 in increments of 0.02 and the best fit to the historical data was ascertained. See also Figure 2.

Supplementary Table 2. Blended trait vs. two-trait models: values of frequency-dependent and direct selection, and evaluation of models.

	**Conformity (β)**	**Selection (σ)**	**MLE**	**AIC**	**ΔAIC**	**weight**
**Blended trait**	0.99	1.71	-20.6	45.2	0	0.750
**Two-trait**	1.28	1.72	-21.7	47.4	2.2	0.250

These models ask whether birds singing both high note clusters and click trains are best represented as having a single, blended trait or two separate traits. All models assumed two innovators joining the population in 1983. See also Supplementary Figure 2.

Supplementary Table 3. Values of frequency-dependent and direct selection parameters, maximum log likelihood, and evaluation of models with different years for the introduction of the click train innovation.

Year of innovation	Conformity (β)	Selection (σ)	MLE	AIC	ΔAIC	weight
**1983**	0.99	1.71	-20.6	45.2	0.0	0.568
**1984**	0.98	1.74	-21.3	46.6	1.4	0.282
**1985**	0.98	1.77	-22.2	48.4	3.2	0.115
**1986**	0.97	1.81	-23.5	51.0	5.8	0.031
**1987**	0.95	1.86	-25.5	55.0	9.8	0.004

Later introductions of the innovation result in decreasing fit of the model to the data, as log likelihood values become increasingly negative. Varying the year when the innovation first appeared did not appreciably change the overall result: a weak frequency-dependent selection effect ($0.95 < \beta < 0.99$, close to but below the neutral value of 1 = a very weak rare-form bias) and moderate to strong direct selection for click trains ($1.71 < \sigma < 1.86$). See also Supplementary Fig. 3.

Supplementary Table 4. Values of frequency-dependent and direct selection, and the log likelihood for best-fit models with different values for the number of click train innovators.

No. innovators (% of pop.)	Conformity (β)	Selection (σ)	MLE	AIC	ΔAIC	weight
**1** (1.4%)	0.98	1.75	-21.8	47.6	9.8	0.0006
**2** (2.9%)	0.99	1.71	-20.6	45.2	5.6	0.0194
**3** (4.3%)	1.02	1.69	-19.8	43.6	4.0	0.0432
**4** (5.7%)	1.04	1.68	-19.3	42.6	3.0	0.0712
**5** (7.1%)	1.05	1.67	-18.7	41.4	1.8	0.1297
**6** (8.6%)	1.06	1.66	-18.4	40.8	1.2	0.1751
**7** (10.0%)	1.07	1.65	-18.1	40.2	0.6	0.2364
**8** (11.4%)	1.08	1.64	-17.8	39.6	0.0	0.3191

Increasing the number of innovators resulted in a better fit of the model to the data. However, direct selection for the novel form (click trains) remained moderate to strong ($\sigma > 1.63$) for all numbers of innovators up to 11.8% of the study site population and frequency dependent selection remained weak ($\beta \approx 1.0$) for all values of innovators tested. We chose to use a value of 2 innovators (a mutation rate of 2.9%) because a) it fits with our long-term recordings of changes in other song features within this population and b) because in studies of bird songs as well as other systems have suggested that an innovation rate, or "cultural mutation rate", of 2% to 5% is realistic[8-10] (see Methods). Despite the differences in model fit, which were small, varying the number of innovators did not change the overall result: all of best-fitting models had weak frequency-dependent selection ($0.98 < \beta < 1.08$) and moderate to strong direct selection for click trains ($0.98 < \sigma < 1.08$). See also Supplementary Figure 4.

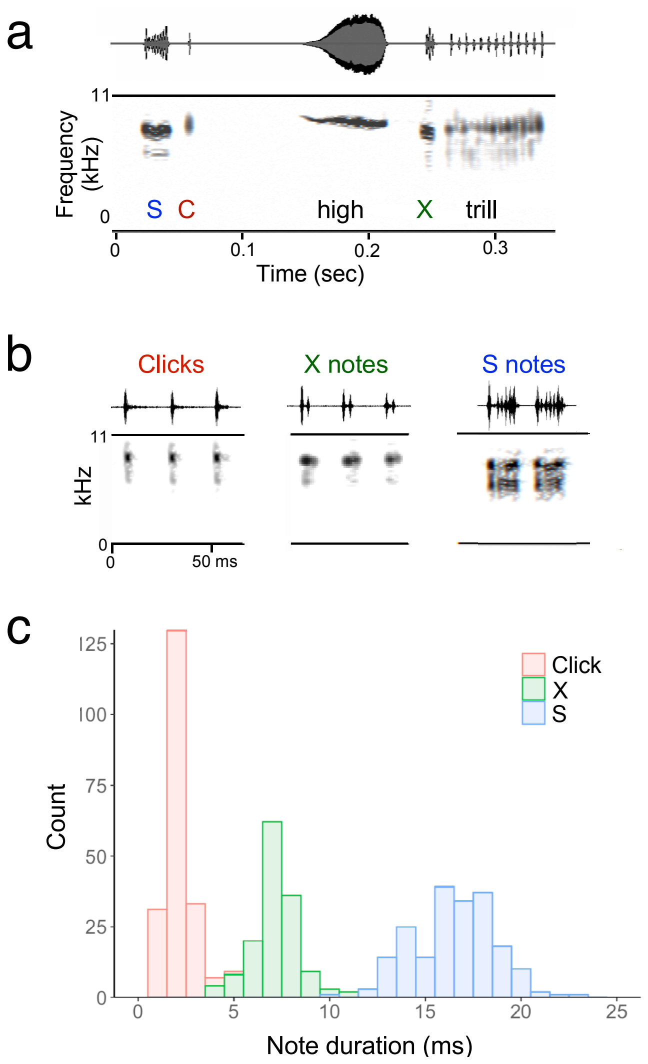

Supplementary Figure 1. Note types found in high note clusters and click trains.
a, Waveform and sound spectrogram of a high note cluster from a 1980 song, including the three typical segments: variable notes, high note, and trill. A combination of one or more types of variable notes (S, X, and Click) form the first part of the high note cluster. The central high note has a mean frequency similar to that of an introductory note, but lacks the sharp frequency modulation of introductory notes. The third component of the high note cluster often included a trill, a series of repeated notes similar to clicks but more closely spaced (3-4 ms) than click notes in a click train (minimum of 9 ms). Here, the trill is preceded by an X note. **b,** Variable notes fall into three classes: S notes, X notes, and Clicks (C). “Click” or “C” notes have a single amplitude peak and are shorter than, “X” notes, which have either two or three amplitude peaks, and are in turn shorter than “S” notes, which have more than four amplitude peaks. **c,** The difference in the number of amplitude peaks of S, X, and Click notes is reflected in their non-overlapping duration distributions: clicks are 1-3 ms long (mode = 2 ms), X notes have a duration of 5-9 ms (mode = 7 ms), while S notes are 13-20 ms long, with most falling between 16 and 18 ms (mode = 16 ms).

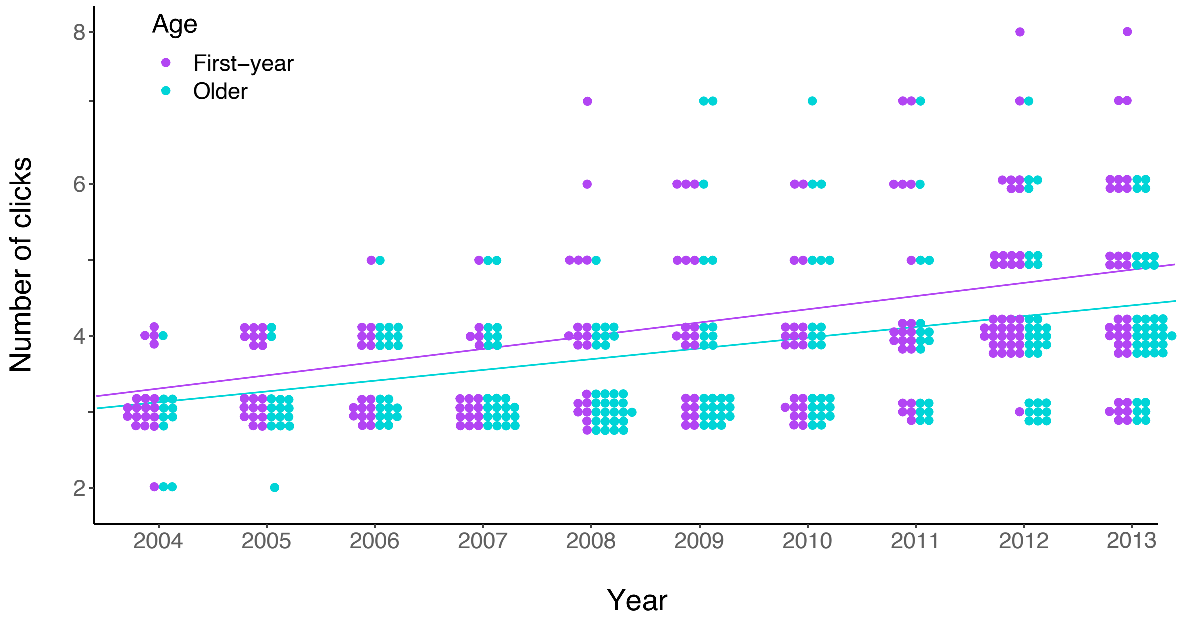

Supplementary Figure 2. Number of clicks in the songs of first-year and older breeding males. Best fit lines for each age class were determined by linear models that included year as well as age class. Most birds retained the same number of clicks in their songs throughout their lifespan, but there were 8 year-to-year changes (4 increases and 4 decreases) after the first year. One of these was a 7-click song sung by an older bird in 2009; the same male sang 5 clicks in 2008 and 6 clicks in 2010. Source data are provided in the Source Data file.

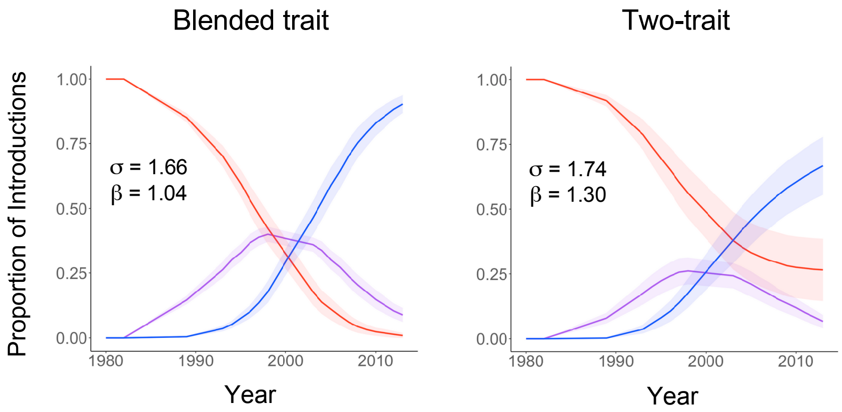

Supplementary Figure 3. The valence of traits in songs with both click trains and high note clusters. Birds singing both click trains and high note clusters could be modeled as having a single, blended trait (left panel) or two separate traits (right panel). Each plot shows the trajectories (mean and 95% confidence interval) of song feature proportions for 50 runs, using the frequency-dependent (β) and direct selection (σ) parameters within the range of 0.5 to 2.0 that resulted in the best fit to the historical data (as defined by the maximum log likelihood). See also Extended Data Table 2.

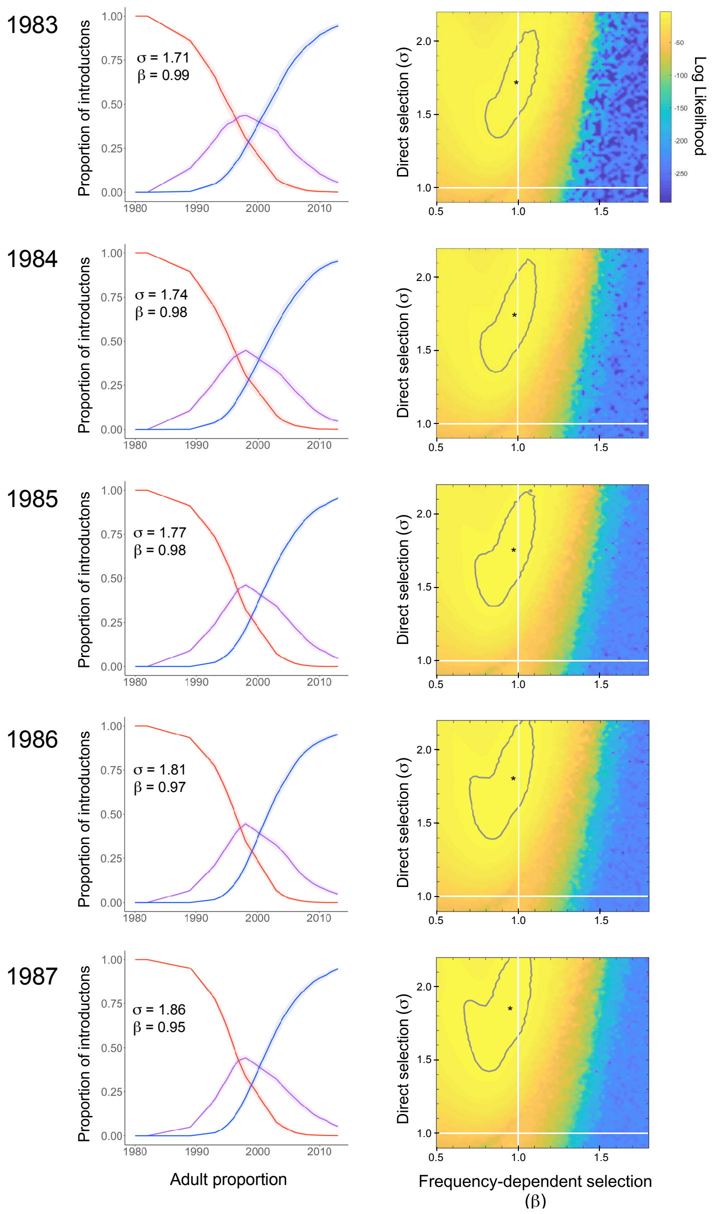

Supplementary Figure 4. Modeling different timing for the introduction of click trains. Click trains were absent in 1982 and present in 1988, and so we modeled introduction for each of the intervening years. For each year, the modeled trajectory of click train adoption is shown in the left panel for the frequency-dependent and direct selection parameters of the model that best fit the historical data (error bands represent 95% confidence intervals). The right panel shows the heat map for frequency-dependent selection values ranging from β = 0.5 (rare form bias) to β = 2.0 (common form bias) and for direct selection values ranging from σ = 0.8 (weak negative selection) to σ = 2.4 (strong positive selection). In each heat map, the neutral values (1.0) are shown with white lines, the parameters that yielded the best fit (ascertained by maximum log likelihood) with an asterisk, and the 95% confidence intervals with a grey contour. See also Supplementary Table 3.

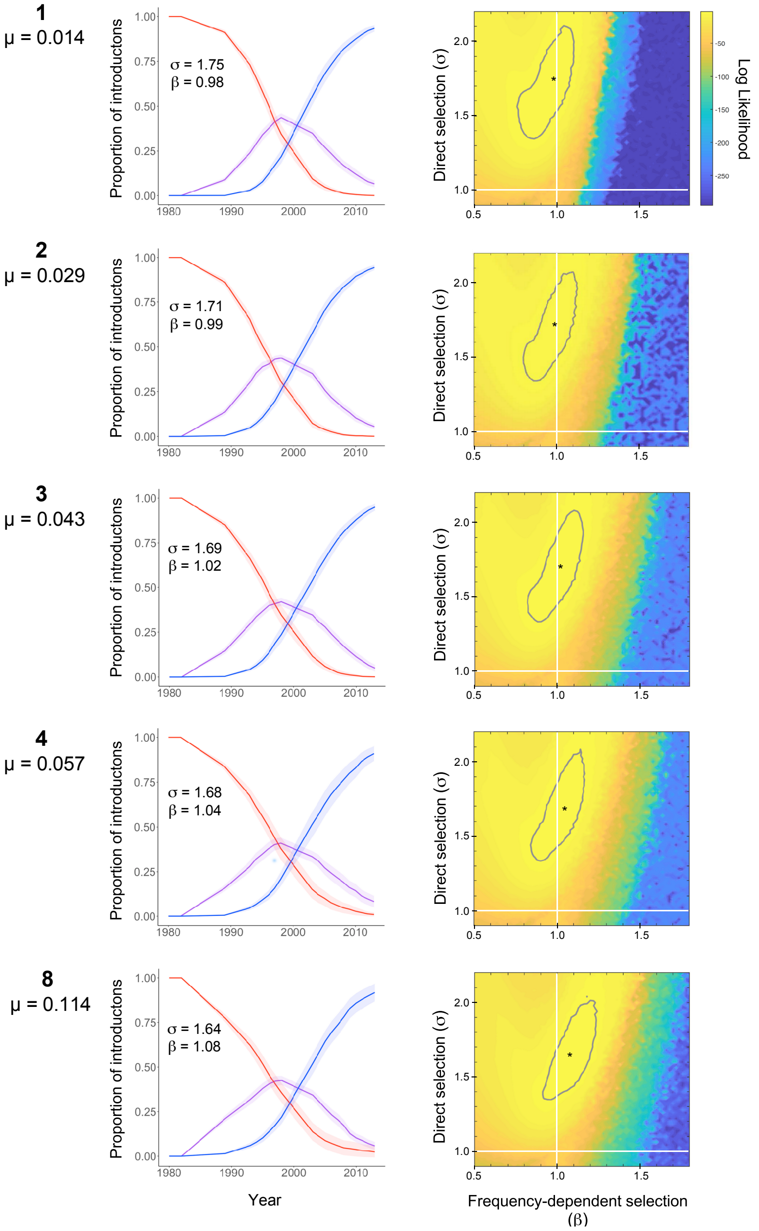

Supplementary Figure 5. Modeling different numbers of innovators. As we do not know how many innovators initially introduced click trains, we ran the model of innovators. Here we show the results for 1 (innovation rate of 0.014), 2, 3, 4 and 8 birds (innovation rate of 0.114), all first appearing in 1983. For each case, the trajectory of click train adoption is shown in the left panel for the parameters of the model that best fit the data (error bands represent 95% confidence intervals). The right panel shows the heat map for frequency-dependent selection (β) values ranging from 0.5 (rare form bias) to 2.0 (common form bias) and for direct selection (σ) values ranging from 0.8 (weak negative selection) to 2.4 (strong positive selection). In each heat map, the neutral values (1.0) are shown with white lines, the parameters that yielded the best fit with an asterisk, and the 95% confidence intervals with a gray contour. See also Supplementary Table 4.

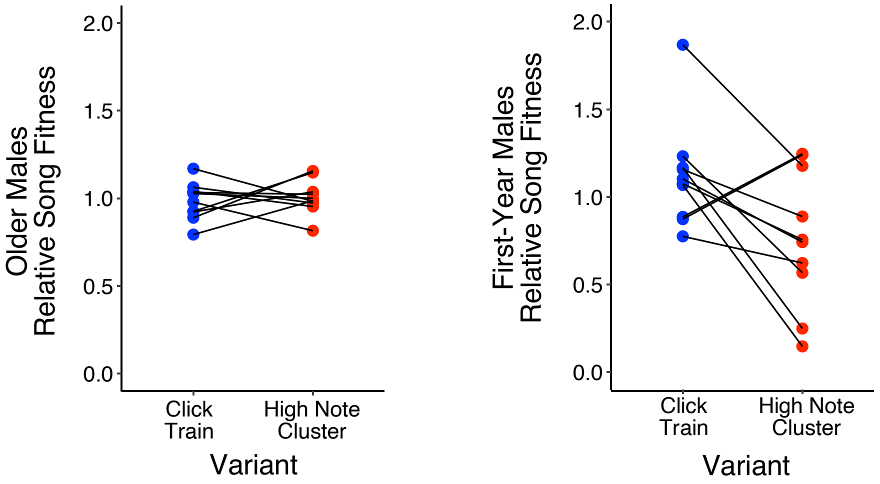

Supplementary Figure 6. Relative fitness of click trains and high note clusters in adult birds and in first-year males. Relative fitness was calculated as the proportion of surviving birds in an age class singing the trait relative to the proportion of all birds singing that trait the previous year. Lines connect the relative fitness of high note clusters and click trains for each year. Source data are provided in the Source Data file.

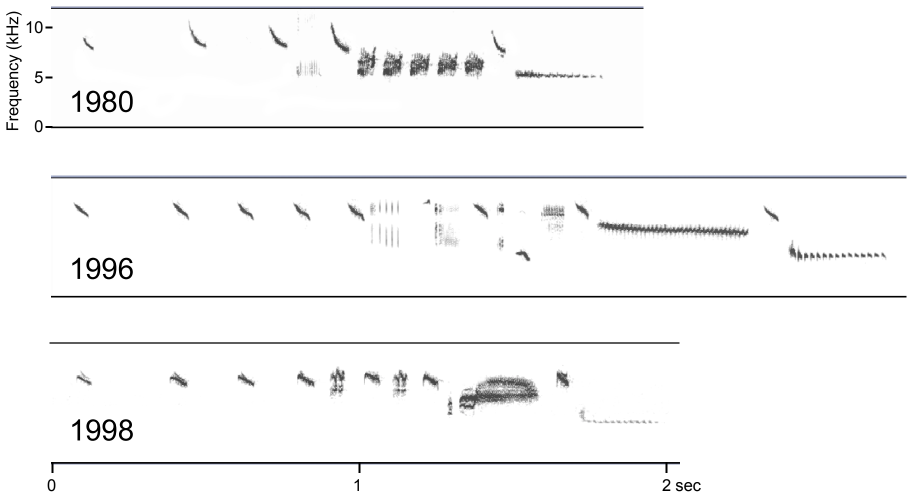

Supplementary Figure 7. Unusual songs recorded on Kent Island. If birds were not banded as nestlings or fledglings, their origin is unknown. Most such birds sing typical Kent Island songs, but some songs included unusual features and might reflect the influence of immigrant birds or songs. Among the 306 songs recorded on Kent Island before 2011 (when the high note cluster had disappeared), three had two or more unusual features not seen in other Kent Island songs. The song recorded in 1980 lacked a buzz segment and had an unusual low trill in place of the high note cluster seen in other songs that year (the multiple repeated notes in the middle section occurred in other Kent Island songs). The song recorded in 1996 had two trills, a short buzz, and unusual middle notes, but also featured a typical Kent Island high note cluster. The song recorded in 1998 had several unusual features, including W-shaped interstitial notes, that did not occur in any other song recorded on Kent Island.
