# Supplementary material for: Cumulative cultural evolution and mechanisms for cultural selection in wild bird songs
Source: Nat Commun. 2022 Jul 11;13:4001. doi: 10.1038/s41467-022-31621-9 (PMC9276793; doi:10.1038/s41467-022-31621-9)
Supplement: Supplementary file 3 — Reporting Summary [file 41467_2022_31621_MOESM3_ESM.pdf]

nature portfolio

Corresponding author(s): Heather Williams

Last updated by author(s): Jun 14, 2022

Reporting Summary

Nature Portfolio wishes to improve the reproducibility of the work that we publish. This form provides structure for consistency and transparency in reporting. For further information on Nature Portfolio policies, see our Editorial Policies and the Editorial Policy Checklist.

Statistics

For all statistical analyses, confirm that the following items are present in the figure legend, table legend, main text, or Methods section.

n/a	Confirmed	
☐	☒	The exact sample size (n) for each experimental group/condition, given as a discrete number and unit of measurement
☐	☒	A statement on whether measurements were taken from distinct samples or whether the same sample was measured repeatedly
☐	☒	The statistical test(s) used AND whether they are one- or two-sided<br>*Only common tests should be described solely by name; describe more complex techniques in the Methods section.*
☐	☒	A description of all covariates tested
☐	☒	A description of any assumptions or corrections, such as tests of normality and adjustment for multiple comparisons
☐	☒	A full description of the statistical parameters including central tendency (e.g. means) or other basic estimates (e.g. regression coefficient) AND variation (e.g. standard deviation) or associated estimates of uncertainty (e.g. confidence intervals)
☐	☒	For null hypothesis testing, the test statistic (e.g. F, t, r) with confidence intervals, effect sizes, degrees of freedom and P value noted<br>*Give P values as exact values whenever suitable.*
☒	☐	For Bayesian analysis, information on the choice of priors and Markov chain Monte Carlo settings
☒	☐	For hierarchical and complex designs, identification of the appropriate level for tests and full reporting of outcomes
☒	☐	Estimates of effect sizes (e.g. Cohen's d, Pearson's r), indicating how they were calculated

Our web collection on statistics for biologists contains articles on many of the points above.

Software and code

Policy information about availability of computer code

Data collection	No software was used for data collection.
Data analysis	SoundEdit Pro (Macromedia)<br>Audacity (www.audacityteam.org)<br>RStudio (version 2022.02.0)<br>R software environment (version 4.1.2)<br>ggplot2 (version 3.3.5)<br>lme4 (version 1.1-27.1)<br>POMP package for R (version 1.1.7)<br>an R script developed by the authors for implementing the song transmission model within POMP, available from github:<br>https://github.com/HeatherWilliams52/SAVSintromodeling

For manuscripts utilizing custom algorithms or software that are central to the research but not yet described in published literature, software must be made available to editors and reviewers. We strongly encourage code deposition in a community repository (e.g. GitHub). See the Nature Portfolio guidelines for submitting code & software for further information.

Data

Policy information about availability of data

All manuscripts must include a data availability statement. This statement should provide the following information, where applicable:

- Accession codes, unique identifiers, or web links for publicly available datasets
- A description of any restrictions on data availability
- For clinical datasets or third party data, please ensure that the statement adheres to our policy

The data set used in the modeling study, a table showing the number of birds singing each type of song in each study year, is provided with the R script, and is available from github at https://github.com/HeatherWilliams52/SAVSintromodeling
Song recordings and the scientific data used to generate Fig. 3a, Fig. 4 and Extended Data Figs. 2 and 6 are available at Dryad: https://doi.org/10.5061/dryad.k98sf7m7x

Field-specific reporting

Please select the one below that is the best fit for your research. If you are not sure, read the appropriate sections before making your selection.

☐ Life sciences ☐ Behavioural & social sciences ☒ Ecological, evolutionary & environmental sciences

For a reference copy of the document with all sections, see nature.com/documents/nr-reporting-summary-flat.pdf

Ecological, evolutionary & environmental sciences study design

All studies must disclose on these points even when the disclosure is negative.

Study description	Long term-recording (30+ years) of the songs of individually identified wild male Savannah sparrows; analysis of those songs; dynamic discrete time modeling of changes observed in the songs over time; playback study testing wild birds' responses to variation of song characteristics.
Research sample	A population of colour-banded Passerculus sandwichensis, at the Bowdoin College Scientific station on Kent Island, NB, Canada.<br>A population of 30-70 males and 30-80 females held territories in the study area. Birds were individually colour banded so they could be recognized in the field.<br>Pairs present in the summer of 2011 were used for the playback study; 25 playbacks were successful (birds were present for the entire duration of the presentation of stimuli).
Sampling strategy	The goal of the study was to band all of the adults present on the study site and record the songs of all the known males on the study site. On average, we recorded 96% of the males on the study site; those we did not record did not sing frequently or left the site.
Data collection	Songs were recorded by a number of different individuals over the years, and equipment varied. See Wheelwright et al., 2008, for details of song recording.<br>The playback experiment was run by two individuals: an observer (H.W.) and a recorder (an undergraduate field assistant). The observer dictated observational data, and the recorder noted the data using pen and paper (see Methods for specifics), tracked the timing, and controlled the presentation of stimuli.
Timing and spatial scale	Songs were recorded between April and July of each year. Songs were recorded in 1980, 1982, 1988-9, 1993-8, and 2003-13.<br>We did not include data after 2013 because of we began an experimental field tutoring study in the summer of 2013.<br>The study area covered 10 hectares.
Data exclusions	We did not include data after the breeding year 2013 because we began an experimental field tutoring study in the summer of 2013.
Reproducibility	When birds were present in more than one year, songs were recorded each year to ensure that they had in fact crystallized and been correctly attributed.<br>Models were run multiple times with stochastically varied parameters to ensure that the modeling results were reproducible.<br>The playback experiment used a total of 25 trials, a relatively large sample size for such studies.
Randomization	For the playback experiment, the order of stimulus delivery was pre-determined so that possible combinations were used.<br>The order for with which birds were used as subjects was determined by past history; adjacent territories were not used on the same or successive days, and birds' presence on the territories was unpredictable and introduced a random element.
Blinding	For the playback experiment, the observer was blind to the stimulus being used.<br>(The person recording the observations also controlled the stimulus delivery.)

Did the study involve field work? ☒ Yes ☐ No

Field work, collection and transport

Field conditions	Temperate northeastern North American grassland and trees on an island in the Bay of Fundy.

	Summer temperatures are moderated by the maritime environment.
Location	Kent Island, New Brunswick, Canada; 44.5818°N, 66.7547°W
Access & import/export	Pathways were created within the habitat and used by humans to avoid disturbing nesting birds.<br>No samples were imported or exported.
Disturbance	Birds were captured, measured, banded and then immediately released onto their territories.<br>Playback experiments simulated a territorial intrusion, and so care was taken to avoid giving birds repeated experience with the stimuli, which might have stressful<br>All animal procedures were carefully reviewed by the Williams College IACUC (WH-D), the Bowdoin College Research and Oversight Committee (2009-18), and the University of Guelph Animal Care Committee (08R601), and were carried out as specified by the Canadian Wildlife Service (banding permit 10789D).

Reporting for specific materials, systems and methods

We require information from authors about some types of materials, experimental systems and methods used in many studies. Here, indicate whether each material, system or method listed is relevant to your study. If you are not sure if a list item applies to your research, read the appropriate section before selecting a response.

Materials & experimental systems

n/a	Involved in the study
☒	☐ Antibodies
☒	☐ Eukaryotic cell lines
☒	☐ Palaeontology and archaeology
☐	☒ Animals and other organisms
☒	☐ Human research participants
☒	☐ Clinical data
☒	☐ Dual use research of concern

Methods

n/a	Involved in the study
☒	☐ ChIP-seq
☒	☐ Flow cytometry
☒	☐ MRI-based neuroimaging

Animals and other organisms

Policy information about studies involving animals; ARRIVE guidelines recommended for reporting animal research

Laboratory animals	NA
Wild animals	Up to 150 adult Savannah sparrows (Passerculus sandwichensis), 30-70 males and 30-80 females, were subjects of the study each year (although only males sing and were recorded, females were also captured and banded so they could be individually identified). Birds were captured with mist nets, which were placed near the target bird's normal route of travel; a playback of a conspecific song was sometimes used to lure the bird into the net. Birds caught in the net were removed within a few minutes and placed in a cloth bag for weighing and while not being handled. Birds usually rest calmly inside these bags, and if they move, they cannot be injured. This holding method appears to substantially reduce stress. Cloth bags are washed frequently.<br>Each bird was banded with four bands; three split plastic color bands and one aluminum USFWS band. Banding followed standard methods used by the Savannah sparrow project participants and originally developed by Nat Wheelwright of Bowdoin College. All birds were measured using rulers and calipers: wing length, tail length, tarsus length, bill length, bill width, and bill depth. Birds were released at the location of capture after processing was completed - within a maximum of 30 minutes.<br>Records from Kent Island were merged into the Kent Island database managed by Ryan Norris of the University of Guelph and submitted to the Canadian Wildlife Service.<br>Playbacks to adult birds in the field did not involve handling. Care was taken in the positioning of the playback speaker and in the timing of playbacks to ensure that the behavioral challenge that a playback represents did not result in excessive stress for the territory holder.
Field-collected samples	NA
Ethics oversight	All animal procedures were carefully reviewed by the Williams College IACUC (WH-D), the Bowdoin College Research and Oversight Committee (2009-18), and the University of Guelph Animal Care Committee (08R601), and were carried out as specified by the Canadian Wildlife Service (banding permit 10789D).

Note that full information on the approval of the study protocol must also be provided in the manuscript.
